# The association of non-exercise estimated cardiorespiratory fitness with hypertension and all-cause mortality in American and Chinese populations: evidence from NHANES and CHARLS

**DOI:** 10.3389/fcvm.2025.1497292

**Published:** 2025-04-15

**Authors:** Mo-Yao Tan, Ping Zhang, Si-Xuan Zhu, Shan Wu, Ming Gao

**Affiliations:** ^1^ Department of Cardiology, Chengdu Integrated TCM and Western Medicine Hospital, Chengdu, Sichuan, China; ^2^ Clinical Medical School, Chengdu University of Traditional Chinese Medicine, Chengdu, Sichuan, China

**Keywords:** non-exercise estimated cardiorespiratory fitness, hypertension, all-cause mortality, NHANES, CHARLS

## Abstract

**Background:**

The Non-Exercise Estimated Cardiorespiratory Fitness (NEE-CRF) method has gained attention in recent years due to its simplicity and effectiveness. Hypertension and all-cause mortality are significant public health issues worldwide, highlighting the importance of exploring the association between NEE-CRF and these two conditions.

**Methods:**

The data from the National Health and Nutrition Examination Survey (NHANES) and the China Health and Retirement Longitudinal Study (CHARLS) were utilized to validate the association between NEE-CRF and hypertension as well as all-cause mortality. NEE-CRF was calculated using a sex-specific longitudinal non-exercise equation. To investigate the relationship between hypertension and all-cause mortality, multivariable regression analysis, generalized additive models, smooth curve fittings, and threshold effect analysis were employed. Logistic regression was used for hypertension analysis, while Cox proportional hazards regression was applied for all-cause mortality. Additionally, we conducted stratified analyses and interaction tests among different groups.

**Results:**

In the NHANES, after fully adjusting for covariates, each unit increase in NEE-CRF was associated with a 24% reduction in the risk of hypertension (OR: 0.76, 95% CI: 0.74–0.78) and a 12% reduction in the risk of all-cause mortality (HR: 0.88, 95% CI: 0.79–0.86). Subgroup analyses showed that the relationship between NEE-CRF and both hypertension and all-cause mortality remained negatively correlated across different subgroups. The negative association was also validated in the CHARLS.

**Conclusions:**

Higher NEE-CRF levels may reduce the risk of developing hypertension and all-cause mortality.

## Introduction

Cardiorespiratory fitness (CRF) is a recognized health indicator that refers to the ability of the lungs, heart, and skeletal muscles to acquire and utilize oxygen during physical activity, typically quantified by maximal oxygen consumption (VO2 max) (1). However, in clinical practice, standardized exercise tests to assess CRF are challenging for individuals with limited mobility and require specialized equipment and personnel, leading to increased costs (2). As a result, Jackson et al. proposed a method for estimating non-exercise cardiorespiratory fitness (NEE-CRF), which has gradually become a widely accepted approach for evaluating CRF in recent years (3). The predictive equation for NEE-CRF incorporates gender, age, easily obtainable physical measurements, and self-reported variables (4). The American Heart Association has noted that NEE-CRF can serve as an alternative to standardized exercise tests for CRF when exercise-based predictions are not sufficiently accurate (5). Numerous studies have explored the association between NEE-CRF and various diseases. For instance, research by Robert A. and colleagues consistently found that higher NEE-CRF is negatively correlated with metabolic risk (6). Some researchers have highlighted a strong association between higher NEE-CRF and a lower incidence of type 2 diabetes (7). Furthermore, a study demonstrated that NEE-CRF can effectively predict mortality even in the absence of actual exercise testing (8). The ability of NEE-CRF to assess metabolic diseases and mortality underscores its significant research potential for further exploration of its relationship with hypertension and all-cause mortality.

Hypertension is a common chronic disease associated with the regulation of the endocrine system, microcirculation, and large arteries (9). Hypertension often contributes to the development of various cardiovascular diseases, stroke, and heart and kidney failure (10). Globally, it is the third leading cause of death, with approximately 12.5% of the global population (one in every eight individuals) affected (11). According to reports from the World Health Organization, approximately 40% of individuals aged 25 and older have hypertension (12). Despite the rising prevalence of hypertension, the rates of control and treatment remain inadequate, placing a significant economic burden on countries (13). All-cause mortality is defined as the total number of deaths from all causes in a given population over a specified period, typically expressed as deaths per 1,000 or 100,000 individuals per year (14). For example, in 2022, cardiovascular diseases caused 19.8 million deaths worldwide (15), while cancer mortality rates have also continued to rise (16). Therefore, using accurate and easily obtainable health parameters like NEE-CRF to monitor the incidence of hypertension and all-cause mortality is of great value.

Currently, studies that simultaneously examine the relationship between NEE-CRF and both hypertension and all-cause mortality are limited. Systematic analyses that combine multiple databases are still scarce, particularly those focusing on different countries and populations. To address this research gap, our study utilized two authoritative public databases—the China Health and Retirement Longitudinal Study (CHARLS) and the US National Health and Nutrition Examination Survey (NHANES)—to systematically analyze the relationship between NEE-CRF, hypertension, and all-cause mortality in both American and Chinese populations.

## Methods

### Study population

This study includes participants from two databases, NHANES and CHARLS. NHANES is a nationwide cross-sectional survey conducted since the 1960s to assess the health and nutritional status of Americans. This survey employs a complex multistage sampling method to select nationally representative samples regularly, supported by the Centers for Disease Control and Prevention (CDC), with data released every 2 years. All study protocols must be approved by the National Center for Health Statistics Ethics Review Board, and informed consent is required from all participants (17).

**Figure 1:**
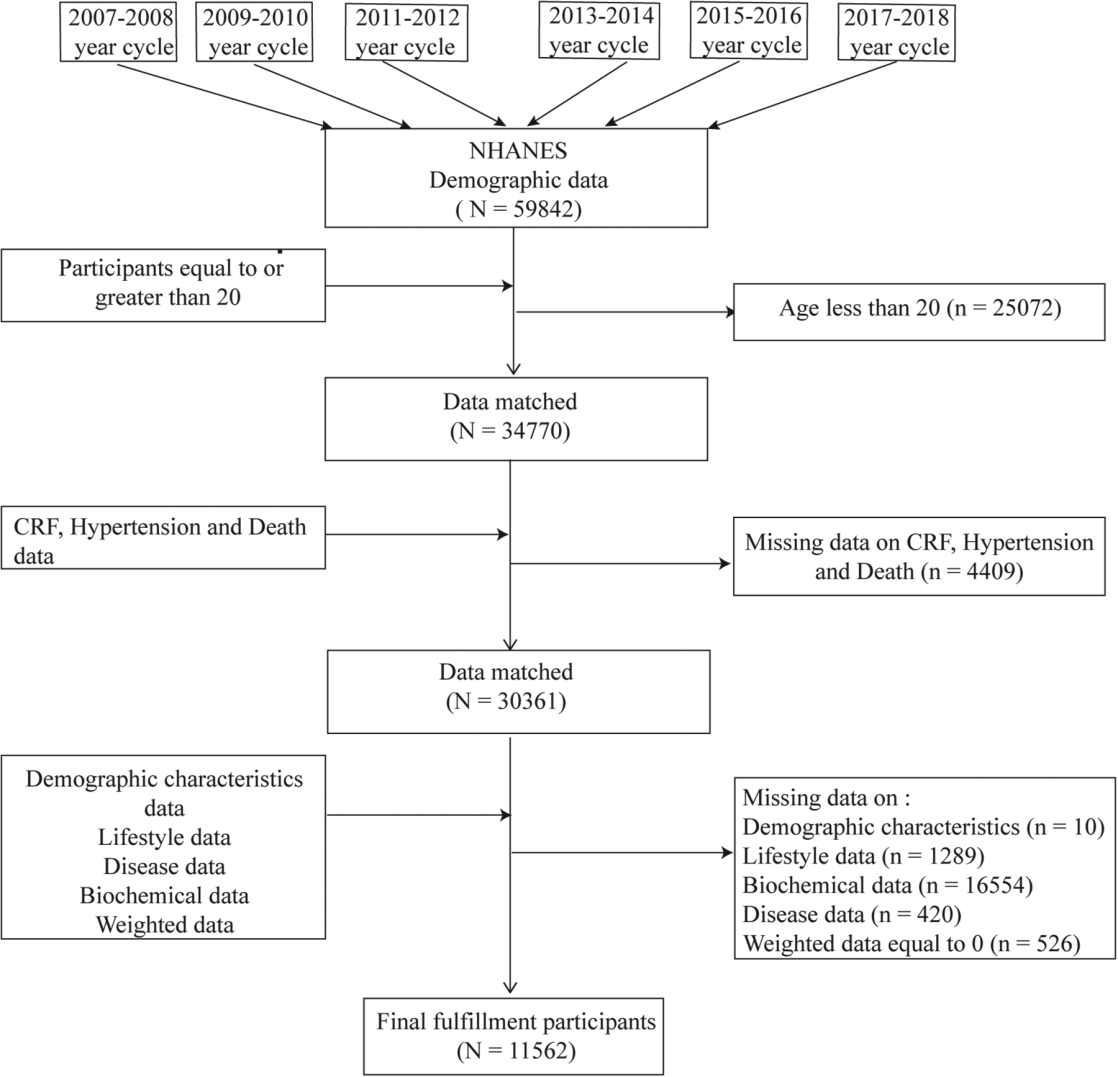
Flowchart of the sample selection from the 2007–2018 National Health and Nutrition Examination Survey (NHANES).

We included data from six cycles between 2007 and 2018, totaling 59,842 individuals. First, we excluded participants younger than 20 years old (*n* = 25,072). Next, participants with missing data on CRF, hypertension, and death were excluded (*n* = 4,409). Subsequently, we excluded participants with missing demographic data (*n* = 10), missing lifestyle data (*n* = 1,289), missing biochemical data (*n* = 16,554), missing disease data (*n* = 420), and those with a weight of zero (*n* = 526). Finally, 11,562 eligible participants were included (Figure 1). The other part of the data comes from CHARLS. Detailed information on the CHARLS database and the screening process can be found in Supplementary Material 1.

### Definition of NEE-CRF

We used the gender-specific body mass index (BMI) model proposed by Jackson et al. (3) to estimate NEE-CRF in metabolic equivalents (METs). The model formulas are as follows: For males: NEE-CRF = 21.2870 + (age × 0.1654) − (age² × 0.0023) − (BMI × 0.2318) − (waist circumference × 0.0337) − (resting heart rate × 0.0390) + (active physical activity × 0.6351) − (smoker × 0.4263). For females: NEE-CRF = 14.7873 + (age × 0.1159) − (age² × 0.0017) − (BMI × 0.1534) − (waist circumference × 0.0088) − (resting heart rate × 0.0364) + (physical activity × 0.5987) − (smoker × 0.2994).

### Definition of hypertension and all-cause mortality

For NHANES and CHARLS, hypertension is diagnosed based on the following criteria (18, 19): an average systolic blood pressure (SBP) of 140 mmHg or more, an average diastolic blood pressure (DBP) of 90 mmHg or more, self-reported hypertension, or the use of prescribed antihypertensive medication.

All-cause mortality in NHANES was identified using National Death Index (NDI) records up to December 31, 2019. Specific cause mortality was determined by ICD-10 codes in the same NDI records. For CHARLS's definition of all-cause mortality, refer to Supplementary Material 2.

### Covariates

Based on previous studies (18), the covariates used for hypertension research utilizing the NHANES database include age (years), sex (male, female), race (mexican American, non-Hispanic Black, non-Hispanic White, other Hispanic, other Race - Including Multi-Racial), education level [Less than 9th grade/9–11th grade (Includes 12th grade with no diploma)/High school/College and Higher], marital status (married/non-married), smoking (former/never/now), physical activity (no/yes), alcohol consumption (no/yes), blood glucose (mmol/L), creatinine (µmol/L), serum uric acid (µmol/L), total cholesterol (TC, mmol/L), high-density lipoprotein cholesterol (HDL-C, mmol/L), low density lipoprotein cholesterol (LDL-C, mmol/L), triglycerides (TG, mmol/L), weight (kg), BMI (kg/m^2^), waist circumference (cm), diabetes (no, yes), and stroke (no, yes) (see Supplementary Material 3 for details). The covariates for all-cause mortality research with NHANES are detailed in Supplementary Material 4. For the covariates included in CHARLS, refer to Supplementary Materials 5, 6.

### Statistical analyses

This study analyzed data from the NHANES database in the United States and the CHARLS database in China. For the CHARLS database, the 2011 survey data was selected as the baseline and followed up until 2020. For the NHANES database, considering sample weighting is essential. Since NHANES employs a complex, multi-stage probability sampling method to select a representative sample of participants, all our analyses incorporate sample weights to approximate national statistics. Continuous variables are presented as means and standard deviations (SD), and categorical variables are expressed as weighted percentages and counts. Firstly, we assessed group differences using Student's *t*-test for continuous variables and the chi-square test for categorical variables. Before performing the *t*-test, we verified the assumptions of normality using the Shapiro–Wilk test and assessed the homogeneity of variances using Levene's test. Secondly, we evaluated the relationship between NEE-CRF and hypertension and all-cause mortality using weighted multivariable logistic regression models and Cox proportional hazards regression models, respectively. Model 1 is the baseline model with no covariate adjustments. Model 2 adjusts for education level and marital status. Model 3 further adjusts for variables including blood glucose, creatinine, serum uric acid, TC, TG, HDL-C, LDL-C, alcohol consumption, and diabetes. Additionally, we analyzed NEE-CRF in quartiles, calculating effect sizes (OR and HR) and their 95% confidence intervals (CI). Thirdly, we identified particularly vulnerable subpopulations by examining the relationship between NEE-CRF, hypertension, and all-cause mortality in both the NHANES and CHARLS databases. For NHANES participants, subgroup analyses were conducted based on alcohol consumption (no/yes), history of stroke (no/yes), diabetes (no/yes), race (Mexican American/Other Hispanic/Other Race), and marital status (married/non-married). For CHARLS participants, subgroup analyses were conducted based on alcohol consumption (no/yes), dyslipidemia (no/yes), diabetes (no/yes), residence (rural/urban), marital status (married/non-married), and education level (elementary school or below/high school/college or above). Interaction tests were performed in both databases to explore heterogeneity across different subgroups. Fourth, we explored potential non-linear relationships between NEE-CRF and both hypertension and all-cause mortality using generalized additive models (GAM), smooth curve fittings, and threshold effect analysis. The criterion for statistical significance was set at a two-sided *P*-value of less than 0.05. All data analyses were performed using R software (version 4.3.2).

## Results

### Characteristics of the baseline population

Table 1 presents the baseline characteristics of participants stratified by hypertension status. The hypertension group includes 4,879 individuals, while the non-hypertension group comprises 6,683 individuals. The mean age of the hypertension group is 59.27 ± 14.70 years, with males accounting for 50.30% and females for 49.70%. The NEE-CRF value for this group is 7.63 ± 2.27, and the resting heart rate is 71.17 ± 12.20 bpm. In contrast, the mean age of the non-hypertension group is 42.74 ± 15.95 years, with 48.97% males and 51.03% females. The NEE-CRF value for this group is 9.38 ± 2.27, and the resting heart rate is 71.35 ± 11.06 bpm. Compared to the non-hypertension group, the hypertension group has significantly higher values for age, BMI, weight, waist circumference, TC, TG, blood glucose, serum uric acid, creatinine, as well as SBP and DBP, with these differences being statistically significant (*P* < 0.001). As shown in Table 2, the quartile ranges for NEE-CRF are 0.01–6.84, 6.84–8.51, 8.51–10.21, and 10.21–15.39, respectively. Additionally, we observed that participants in the fourth quartile had significantly lower age, resting heart rate, BMI, waist circumference, TC, and HDL-C compared to those with lower NEE-CRF (*P* < 0.001). The basic characteristics of the Chinese population are detailed in Supplementary Materials 7, 8.

**Table 1:** Population characteristics classified by hypertension status in American population.

Variable	Total (*n* = 11,562)	Non-hypertension (*n* = 6,683)	Hypertension (*n* = 4,879)	*P*-value
Age (years)	49.72 ± 17.46	42.74 ± 15.95	59.27 ± 14.70	**<0.001**
rHR (bpm)	71.17 ± 11.50	71.35 ± 11.06	71.17 ± 12.20	0.43
BMI (kg/m^2^)	29.12 ± 6.79	27.85 ± 6.29	30.87 ± 7.07	**<0.001**
Waist circumference (cm)	99.41 ± 16.30	95.42 ± 15.44	104.96 ± 15.85	**<0.001**
Weight (kg)	81.79 ± 21.15	78.54 ± 19.50	86.31 ± 22.48	**<0.001**
NEE-CRF (METs)	8.65 ± 2.43	9.38 ± 2.27	7.63 ± 2.27	**<0.001**
SBP (mmHg)	123.01 ± 18.14	114.68 ± 11.11	134.21 ± 19.87	**<0.001**
DBP (mmHg)	69.55 ± 11.80	67.74 ± 9.72	71.83 ± 13.89	**<0.001**
Glucose (mmol/L)	6.04 ± 1.86	5.71 ± 1.47	6.50 ± 2.22	**<0.001**
Creatinine (µmol/L)	78.08 ± 36.72	73.10 ± 20.55	85.00 ± 50.51	**<0.001**
SUA (µmol/L)	327.01 ± 84.57	311.78 ± 77.92	348.12 ± 88.80	**<0.001**
TG (mmol/L)	1.32 ± 0.74	1.23 ± 0.71	1.43 ± 0.77	**<0.001**
TC (mmol/L)	4.95 ± 1.05	4.93 ± 1.02	4.96 ± 1.08	0.17
HDL-C (mmol/L)	1.40 ± 0.41	1.41 ± 0.40	1.39 ± 0.42	**<0.01**
LDL-C (mmol/L)	2.94 ± 0.91	2.96 ± 0.89	2.92 ± 0.94	**0.03**
Sex, *n* (%)				0.23
Female	5,843 (50.53)	3,395 (51.03)	2,448 (49.70)	
Male	5,719 (49.47)	3,288 (48.97)	2,431 (50.30)	
Ethics, *n* (%)				**<0.001**
Mexican American	1,779 (8.54)	1,201 (10.28)	578 (5.68)	
Non-hispanic black	2,257 (9.88)	1,056 (8.44)	1,201 (12.26)	
Non-hispanic white	4,937 (68.46)	2,788 (67.23)	2,149 (70.48)	
Other hispanic	1,288 (5.83)	790 (6.54)	498 (4.67)	
Other race—including multi-racial	1,301 (7.29)	848 (7.51)	453 (6.91)	
Marital status, *n* (%)				**<0.001**
Married	6,020 (56.21)	3,392 (54.43)	2,628 (59.13)	
Non-married	5,542 (43.79)	3,291 (45.57)	2,251 (40.87)	
Education level, *n* (%)				**<0.001**
Less than 9th grade	1,109 (4.88)	561 (4.27)	548 (5.87)	
9–11th grade (Includes 12th grade with no diploma)	1,611 (10.28)	865 (9.49)	746 (11.57)	
High school	1,005 (7.57)	560 (7.27)	445 (8.06)	
College and higher	7,837 (77.28)	4,697 (78.97)	3,140 (74.50)	
Smoke, *n* (%)				**<0.001**
Former	6,444 (55.55)	3,945 (58.82)	2,499 (50.17)	
Never	2,284 (18.70)	1,401 (19.37)	883 (17.59)	
Now	2,834 (25.75)	1,337 (21.81)	1,497 (32.24)	
Drink, *n* (%)				**0.003**
No	1,593 (10.49)	836 (9.64)	751 (11.89)	
Yes	9,969 (89.51)	5,847 (90.36)	4,122 (88.11)	
Active physical activity, *n* (%)				**0.01**
No	9,221 (77.72)	5,212 (76.48)	4,099 (79.76)	
Yes	2,341 (22.28)	1,471 (23.52)	870 (20.24)	
Death, *n* (%)				**<0.001**
No	10,567 (93.90)	6,411 (96.97)	4,156 (88.85)	
Yes	995 (6.10)	272 (3.03)	723 (11.15)	
Stroke, *n* (%)				**<0.001**
No	11,130 (97.14)	6,588 (98.77)	4,542 (94.44)	
Yes	432 (2.86)	95 (1.23)	337 (5.56)	
DM, *n* (%)				**<0.001**
No	9,157 (84.32)	5,965 (92.21)	3,192 (71.34)	
Yes	2,405 (15.68)	718 (7.79)	1,687 (28.66)	

All values are presented as proportion (%) for categorical variables, assessed via weighted chi-square tests, or mean (standard deviation) for continuous variables, assessed via weighted Student's *t*-tests. NEE-CRF, non-exercise estimated cardiorespiratory fitness; rHR, resting heart rate; SBP, systolic blood pressure; DBP, diastolic blood pressure; SUA, serum uric acid; TG, triglyceride; TC, total cholesterol; HDL-C, high density lipoprotein cholesterol; LDL-C, low density lipoprotein cholesterol; DM, diabetes mellitus.

Bold values indicate statistical significance.

**Table 2:** Population characteristics classified by NEE-CRF four categories in American population.

Characteristics	Cardiorespiratory fitness					*P*-value
	Total	Q1	Q2	Q3	Q4	
	(0.01–15.39)	(0.01–6.84)	(6.84–8.51)	(8.51–10.21)	(10.21–15.39)	
Age (years)	49.72 ± 17.46	62.16 ± 16.34	52.72 ± 16.13	44.36 ± 15.18	39.88 ± 13.20	**<0.001**
rHR (bpm)	71.17 ± 11.50	74.75 ± 12.46	72.16 ± 11.19	70.88 ± 10.56	66.99 ± 10.28	**<0.001**
BMI (kg/m^2^)	29.12 ± 6.79	35.52 ± 7.94	29.78 ± 4.70	26.33 ± 4.54	24.96 ± 3.55	**<0.001**
Waist circumference (cm)	99.41 ± 16.30	114.18 ± 16.75	101.25 ± 12.11	92.73 ± 13.34	89.81 ± 10.15	**<0.001**
Weight (kg)	81.79 ± 21.15	95.66 ± 25.95	81.51 ± 17.84	74.75 ± 18.71	75.66 ± 13.28	**<0.001**
SBP (mmHg)	123.01 ± 18.14	130.15 ± 19.74	124.03 ± 18.21	118.46 ± 17.26	119.51 ± 14.68	**<0.001**
DBP (mmHg)	69.55 ± 11.80	67.97 ± 13.15	69.87 ± 11.58	69.82 ± 11.35	70.50 ± 10.88	**<0.001**
Glucose (mmol/L)	6.04 ± 1.86	6.62 ± 2.20	6.14 ± 2.06	5.72 ± 1.50	5.72 ± 1.46	**<0.001**
Creatinine (µmol/L)	78.08 ± 36.72	79.98 ± 35.34	74.92 ± 38.66	74.40 ± 36.63	84.01 ± 35.56	**<0.001**
SUA (µmol/L)	327.01 ± 84.57	346.72 ± 86.89	316.56 ± 83.09	307.36 ± 87.38	340.86 ± 72.72	**<0.001**
TG (mmol/L)	1.32 ± 0.74	1.47 ± 0.73	1.34 ± 0.73	1.20 ± 0.72	1.26 ± 0.77	**<0.001**
TC (mmol/L)	4.95 ± 1.05	4.96 ± 1.09	5.02 ± 1.06	4.90 ± 1.01	4.90 ± 1.01	**<0.001**
HDL-C (mmol/L)	1.40 ± 0.41	1.38 ± 0.40	1.41 ± 0.41	1.45 ± 0.43	1.36 ± 0.40	**<0.001**
LDL-C (mmol/L)	2.94 ± 0.91	2.91 ± 0.95	3.00 ± 0.91	2.90 ± 0.89	2.96 ± 0.89	
Sex, *n* (%)						**<0.001**
Female	5,843 (50.53)	2,121 (74.78)	1,970 (71.75)	1,585 (57.58)	167 (5.81)	
Male	5,719 (49.47)	750 (25.22)	899 (28.25)	1,299 (42.42)	2,771 (94.19)	
Ethics, *n* (%)						**<0.001**
Mexican American	1,779 (8.54)	367 (6.21)	471 (8.38)	468 (9.04)	473 (10.08)	
Non-hispanic black	2,257 (9.88)	613 (10.79)	600 (11.00)	490 (8.48)	554 (9.49)	
Non-hispanic white	4,937 (68.46)	1,472 (74.89)	1,202 (68.86)	1,149 (67.03)	1,114 (64.28)	
Other hispanic	1,288 (5.83)	283 (4.10)	363 (5.79)	360 (6.89)	282 (6.27)	
Other race—including multi-racial	1,301 (7.29)	136 (4.01)	233 (5.97)	417 (8.56)	515 (9.88)	
Marital status, *n* (%)						**<0.001**
Married	6,020 (56.21)	1,403 (55.04)	1,569 (59.32)	1,591 (58.59)	1,457 (52.21)	
Non-married	5,542 (43.79)	1,468 (44.96)	1,300 (40.68)	1,293 (41.41)	1,481 (47.79)	
Physical activity, *n* (%)						**<0.001**
No	9,221 (77.72)	2,615 (90.36)	2,433 (84.20)	2,294 (77.83)	1,879 (61.79)	
Yes	2,341 (22.28)	256 (9.64)	436 (15.80)	590 (22.17)	1,059 (38.21)	
Education, *n* (%)						**0.002**
College and higher	7,837 (77.28)	1,819 (74.14)	1,924 (76.70)	2,058 (79.94)	2,036 (77.81)	
Elementary school and below	2,720 (15.15)	786 (18.18)	689 (14.80)	597 (12.94)	648 (15.10)	
High school	1,005 (7.57)	266 (7.67)	256 (8.50)	229 (7.12)	254 (7.10)	
Smoke, *n* (%)						**<0.001**
Current smoker	6,444 (55.55)	1,439 (48.32)	1,603 (55.46)	1,719 (58.49)	1,683 (58.68)	
Former smoker	2,284 (18.70)	387 (12.82)	534 (17.66)	553 (18.43)	810 (24.60)	
Never	2,834 (25.75)	1,045 (38.86)	732 (26.88)	612 (23.08)	445 (16.72)	
Drink, *n* (%)						**<0.001**
No	1,593 (10.49)	545 (14.67)	454 (11.95)	354 (9.05)	240 (7.21)	
Yes	9,969 (89.51)	2,326 (85.33)	2,415 (88.05)	2,530 (90.95)	2,698 (92.79)	
Hypertension, *n* (%)						**<0.001**
No	6,683 (62.18)	908 (35.09)	1,489 (56.02)	2,024 (74.15)	2,262 (78.17)	
Yes	4,879 (37.82)	1,963 (64.91)	1,380 (43.98)	860 (25.85)	676 (21.83)	
DM, *n* (%)						**<0.001**
No	9,157 (84.32)	1,729 (65.73)	2,198 (81.99)	2,536 (91.71)	2,694 (94.39)	
Yes	2,405 (15.68)	1,142 (34.27)	671 (18.01)	348 (8.29)	244 (5.61)	
Stroke, *n* (%)						**<0.001**
No	11,130 (97.14)	2,667 (93.72)	2,740 (96.73)	2,817 (98.26)	2,906 (99.19)	
Yes	432 (2.86)	204 (6.28)	129 (3.27)	67 (1.74)	32 (0.81)	

All values are presented as proportion (%) for categorical variables, assessed via weighted chi-square tests, or mean (standard deviation) for continuous variables, assessed via weighted Student's *t*-tests. NEE-CRF, non-exercise estimated cardiorespiratory fitness; rHR, resting heart rate; SBP, systolic blood pressure; DBP, diastolic blood pressure; SUA, serum uric acid; TG, triglyceride; TC, total cholesterol; HDL-C, high density lipoprotein cholesterol; LDL-C, low density lipoprotein cholesterol; DM, diabetes mellitus.

Bold values indicate statistical significance.

### Association between the NEE-CRF with hypertension and all-cause mortality

Tables 3, 4 provide a detailed overview of the relationship between NEE-CRF and hypertension as well as all-cause mortality in the American population. After adjusting for all covariates, each unit increase in NEE-CRF was associated with a 24% reduction in the risk of hypertension (OR = 0.76, 95% CI: 0.74–0.78) and a 12% reduction in mortality (HR = 0.88, 95% CI: 0.79–0.86). When divided into quartiles, the highest quartile group showed a 79% reduction in the risk of developing hypertension (OR = 0.21, 95% CI: 0.18–0.25) and a 74% reduction in mortality (HR = 0.26, 95% CI: 0.18–0.37) compared to the lowest quartile group.

**Table 3:** Associations of NEE-CRF with hypertension in American population.

Hypertension	OR^a^ (95% CI), *P*-value		
	Crude model^b^	Model 1^c^	Model 2^d^
Continuous	0.72 (0.70,0.74) **<0.001**	0.73 (0.71, 0.76) **<0.001**	0.76 (0.74, 0.78) **<0.001**
Categorical			
Q1	Reference	Reference	Reference
Q2	0.43 (0.38,0.49) **<0.001**	0.57 (0.49, 0.66) **<0.001**	0.63 (0.54, 0.74) **<0.001**
Q3	0.20 (0.17,0.23) **<0.001**	0.27 (0.23, 0.32) **<0.001**	0.31 (0.26, 0.37) **<0.001**
Q4	0.16 (0.14,0.18) **<0.001**	0.18 (0.14, 0.22) **<0.001**	0.21 (0.18, 0.25) **<0.001**
*P* for trend	**<0.001**	**<0.001**	**<0.001**

In sensitivity analysis, NEE-CRF is transformed from a continuous variable to a categorical variable (Quartiles). NEE-CRF, non-exercise estimated cardiorespiratory fitness; 95% CI, 95% confidence interval; OR, odds ratio.

Bold values indicate statistical significance.

^a^ OR: effect size.

^b^ Crude model: no covariates were adjusted.

^c^ Model 1: adjusted for education level and marital status.

^d^ Model 2: adjusted for education level, marital status, blood glucose, creatinine, serum uric acid, total cholesterol, triglyceride, HDL-C, LDL-C, alcohol consumption, and diabetes.

**Table 4:** Associations of NEE-CRF with all-cause mortality in American population.

Cardiorespiratory fitness	HR^a^ (95% CI), *P*-value		
	Crude model^b^	Model 1^c^	Model 2^d^
Continuous	0.73 (0.70,0.75) **<0.001**	0.80 (0.76, 0.84) **<0.001**	0.88 (0.79, 0.86) **<0.001**
Categorical			
Q1	Reference	Reference	Reference
Q2	0.40 (0.33,0.48) **<0.001**	0.54 (0.45, 0.67) **<0.001**	0.62 (0.50, 0.76) **<0.001**
Q3	0.21 (0.16,0.27) **<0.001**	0.33 (0.24, 0.45) **<0.001**	0.36 (0.26, 0.50) **<0.001**
Q4	0.14 (0.10,0.19) **<0.001**	0.24 (0.17, 0.35) **<0.001**	0.26 (0.18, 0.37) **<0.001**
*P* for trend	**<0.001**	**<0.001**	**<0.001**

In sensitivity analysis, NEE-CRF is transformed from a continuous variable to a categorical variable (Quartiles). NEE-CRF, non-exercise estimated cardiorespiratory fitness; 95% CI, 95% confidence interval; HR, hazard ratio.

Bold values indicate statistical significance.

^a^ HR: effect size.

^b^ Crude model: no covariates were adjusted.

^c^ Model 1: adjusted for education level and marital status.

^d^ Model 2: adjusted for education level, marital status, blood glucose, creatinine, serum uric acid, total cholesterol, triglyceride, HDL-C, LDL-C, alcohol consumption, and diabetes.

In contrast, as shown in Supplementary Materials 9, 10, among the Chinese population, each unit increase in NEE-CRF is associated with a 13% reduction in the likelihood of developing hypertension (OR = 0.87, 95% CI: 0.81–0.94) and a similar 12% reduction in mortality risk (HR = 0.88, 95% CI: 0.80–0.96). Notably, when comparing the highest to the lowest quartile groups, the risk of hypertension decreased by 48% (OR = 0.52, 95% CI: 0.36–0.75), while mortality was reduced by 45% (HR = 0.55, 95% CI: 0.34–0.90).

### Subgroup and interaction analyses

The results of the subgroup analyses demonstrated that NEE-CRF consistently showed a negative correlation with hypertension and all-cause mortality across different subgroups in both American and Chinese populations (Supplementary Materials 11, 12). Notably, in the American population, factors such as alcohol consumption, stroke, diabetes, and marital status significantly influenced the strength of the association between NEE-CRF and hypertension (all interaction *P*-values <0.05). Similarly, stroke, diabetes, and race had a significant impact on the association between NEE-CRF and all-cause mortality (all interaction *P*-values <0.05). However, in the Chinese population, the associations between NEE-CRF and both hypertension and all-cause mortality remained consistent across subgroups stratified by residence, alcohol consumption, diabetes, dyslipidemia, marital status, and education level (all interaction *P*-values >0.05).

### Nonlinear relationship NEE-CRF with hypertension and all-cause mortality

**Figure 2:**
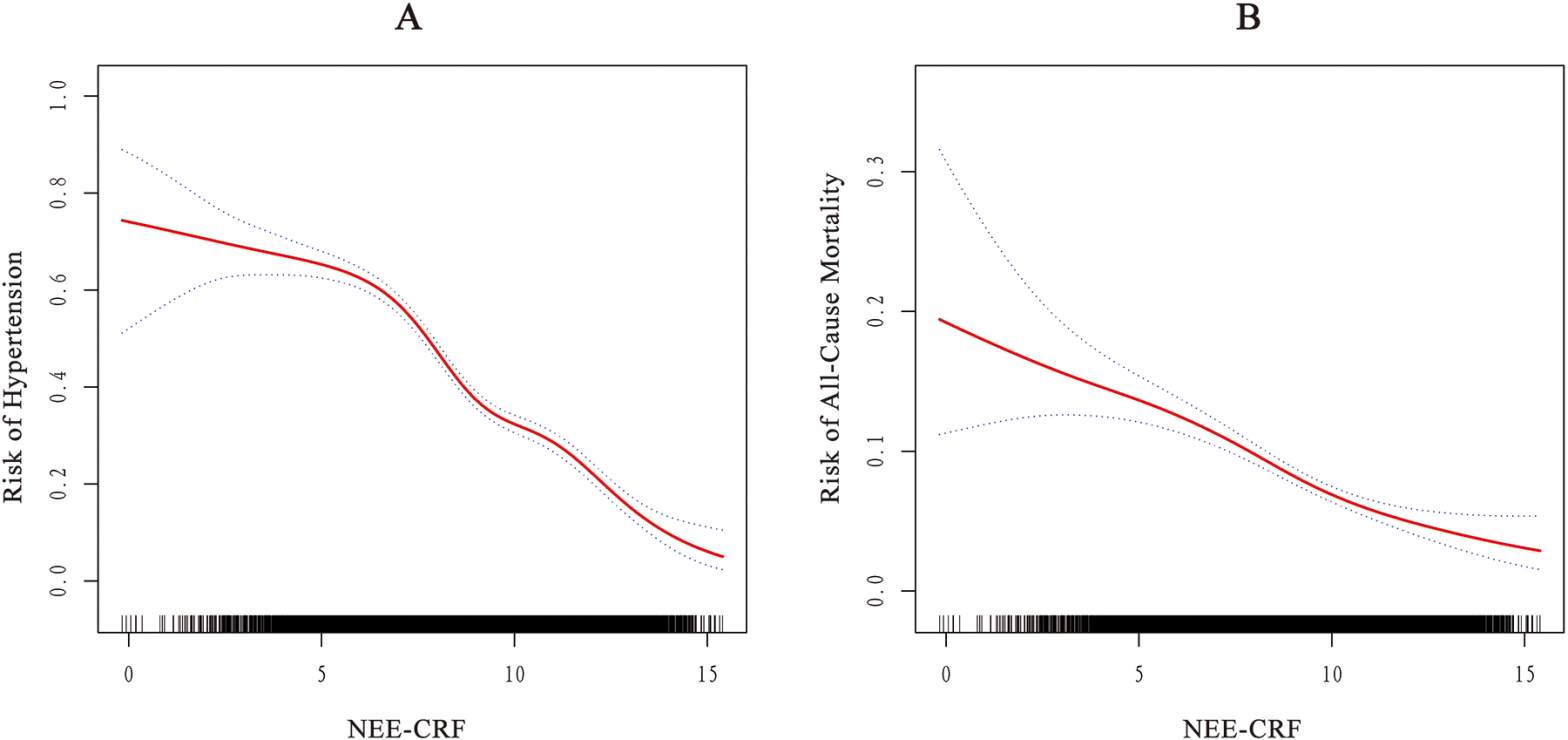
Smoothed curve fitting: dose-response relationship between NEE-CRF with **(A)** hypertension and **(B)** all-cause mortality.

The results of the GAM and smooth curve fitting shown in Figure 2 revealed a nonlinear relationship between NEE-CRF and both hypertension and all-cause mortality in the American population. The threshold analysis found that when NEE-CRF reached 6.00, the risk of hypertension hit a critical point, and when NEE-CRF reached 7.40, there was a significant change in the risk of all-cause mortality (Table 5). Specifically, when NEE-CRF was ≥7.40, each additional unit increase in NEE-CRF was associated with a 20% reduction in the risk of all-cause mortality (HR: 0.80, 95% CI 0.80–0.90). In contrast, when NEE-CRF was <7.40, changes in NEE-CRF were not significantly associated with the risk of all-cause mortality. Additionally, we observed that when NEE-CRF reached 6.00, the risk of hypertension significantly decreased (OR: 0.70, 95% CI 0.70–0.80).

**Table 5:** Threshold effect analysis of NEE-CRF with hypertension and all-cause mortality using two-piecewise linear regression model in American population.

All-cause mortality	Adjust HR (95% CI) *P* value
Fitting by linear regression model	0.90 (0.88, 0.92) <0.001
Fitting by two-piecewise Cox proportional risk model	
Inflection point	7.40
<7.40	0.92 (0.90, 1.00) 0.16
>7.40	0.80 (0.80, 0.90) <0.001
Log likelihood ratio test	0.001
Hypertension	Adjust OR (95% CI) *P* value
Fitting by linear regression model	0.80 (0.70, 0.80) <0.001
Fitting by two-piecewise linear regression model	
Inflection point	6.00
<6.00	0.90 (0.90, 0.99) 0.028
>6.00	0.70 (0.70, 0.80) <0.001
Log likelihood ratio test	<0.001

Adjusted for education level, marital status, blood glucose, creatinine, serum uric acid, total cholesterol, triglyceride, HDL-C, LDL-C, alcohol consumption, and diabetes. NEE-CRF, non-exercise estimated cardiorespiratory fitness; 95% CI, 95% confidence interval; OR, odds ratio; HR, hazard ratio.

In the Chinese population, the results presented in Supplementary Material 13 also revealed a nonlinear relationship between NEE-CRF and both hypertension and all-cause mortality. Supplementary Material 14 indicated that the threshold for the association between NEE-CRF and all-cause mortality was identified as 8.74 METs. Below this threshold, NEE-CRF was significantly negatively correlated with all-cause mortality (HR: 0.66, 95% CI 0.56–0.79), whereas above the threshold, the relationship was not significant (HR: 0.96, 95% CI 0.85–1.09). Furthermore, the log-likelihood ratio test indicated that there was no statistically significant threshold in the association between NEE-CRF and hypertension (*P* > 0.05).

## Discussion

This study, using data from the Chinese CHARLS and American NHANES databases, found that higher levels of NEE-CRF are associated with a lower risk of hypertension and all-cause mortality. These findings carry significant clinical implications. First, in resource-limited clinical settings, healthcare professionals can easily and non-invasively estimate CRF through non-exercise assessment methods, enabling earlier implementation of disease prevention and rehabilitation measures (20). This approach not only streamlines clinical procedures but also facilitates the creation of personalized health management plans for patients (21). Second, for individuals at high risk of hypertension, NEE-CRF-based data can guide healthcare professionals in promptly adjusting lifestyle recommendations, thereby effectively reducing the risk of developing the condition (21). Furthermore, improving NEE-CRF levels can help lower mortality rates from various diseases and enhance patient prognosis (22). Through continuous health management and lifestyle interventions, patients can reduce the likelihood of disease recurrence, extend their life expectancy, and improve their quality of life (22).

Previous studies have explored the relationship between NEE-CRF and both hypertension and all-cause mortality. For instance, in a rural Chinese cohort study, Zhao et al. found that patients with more than a 2% increase in NEE-CRF had a 24% lower incidence of hypertension, while those with more than a 2% decrease in NEE-CRF had a 52% higher risk (23). A follow-up study of American Caucasians indicated that both a high NEE-CRF trajectory and a high average NEE-CRF were associated with a reduced risk of hypertension (21). Similarly, Liu et al.'s study involving 4,286 elderly Chinese individuals showed that those with higher NEE-CRF exhibited better arterial pressure performance, characterized by lower systolic and diastolic blood pressure over time (24). Another study involving 330,769 American retirees demonstrated that higher NEE-CRF was independently associated with a lower risk of mortality (22). Research from England found a consistent association between NEE-CRF and both all-cause and cardiovascular mortality, with a 12%–15% reduction in all-cause mortality for every 1.6–1.7 unit increase in NEE-CRF (20). Additionally, a study on an American population reported that for each 1-MET increase in NEE-CRF, cancer mortality risk decreased by 30% in men and 28% in women (25). Since the parameters used to assess NEE-CRF are often closely related to an individual's lifestyle (e.g., diet, weight), which also significantly influence hypertension, this further underscores the connection between the two (4, 26). Lee et al.'s research pointed out that higher NEE-CRF in middle-aged Europeans was associated with lower subclinical atherosclerosis and vascular stiffness (27), both of which are high-risk factors for the development of hypertension (28). Moreover, studies have shown that good cardiorespiratory fitness implies a stronger ability to adapt to daily activities and environmental changes, thereby reducing mortality risk due to various factors (29, 30). In summary, existing literature confirms that an increase in NEE-CRF can reduce the risk of hypertension and all-cause mortality, which aligns with our study findings.

The relationship between NEE-CRF and reduced all-cause mortality is complex and multifaceted, involving a variety of mechanisms. Some key mechanisms include: Firstly, improving NEE-CRF means the heart can pump blood more efficiently, providing adequate oxygen and nutrients to various parts of the body, thereby reducing the risk of heart disease (31). It also helps lower resting blood pressure, which reduces the incidence of cardiovascular events such as heart attacks and strokes (21). Additionally, enhanced NEE-CRF helps regulate autonomic nervous function, which normalizes the excessive activation of the sympathetic nervous system, thereby lowering the risk of fatal arrhythmias (32). Improved NEE-CRF also combats atherosclerosis and positively influences cardiovascular risk factors such as lipids, blood pressure, glucose, and obesity (33). This not only enhances metabolic health but also promotes lipid metabolism, reducing the risk of diabetes, obesity, and metabolic syndrome (34). Collectively, these benefits contribute to reduced all-cause mortality. Secondly, enhancing NEE-CRF can also improve mental and cognitive health (35). Research shows that higher levels of NEE-CRF are associated with greater gray matter volume in regions of interest related to Alzheimer's disease, reduced white matter hyperintensities, and better cognitive function and emotional states (36). These improvements not only lower the risk of developing Alzheimer's disease and depression but also protect brain structure, thereby promoting overall mental health (37). Improved mental health significantly benefits physical health, ultimately helping to reduce all-cause mortality (38). Regarding hypertension, a common chronic disease, its impact on all-cause mortality involves multiple complex physiological mechanisms and pathological processes, including: Firstly, hypertension can damage vascular endothelium and lead to arteriosclerosis. Over time, arterial walls thicken and lose elasticity, restricting blood flow and increasing the workload on the heart (39). Persistent hypertension may result in left ventricular hypertrophy, potentially progressing to heart failure, which significantly increases the risk of death (40). Additionally, hypertension is a major risk factor for coronary artery disease, leading to severe cardiac events such as angina and myocardial infarction, further raising all-cause mortality (41). Secondly, hypertension increases the risk of stroke (42). Vascular damage caused by hypertension can trigger systemic inflammatory responses, impairing endothelial function and increasing the risk of thrombosis (43). These clots can travel to the brain, causing infarction (44). Long-term hypertension also impacts small cerebral vessels, leading to narrowing, blockage, or bleeding, which increases the risk of stroke (42). Finally, long-term hypertension can cause hardening and narrowing of small renal arteries, affecting the kidneys' blood supply (45). Persistent ischemia and damage to renal tubules can lead to interstitial inflammation and fibrosis, ultimately damaging renal structure and function (46). This can result in serious consequences such as uremia, further increasing all-cause mortality (46).

Our subgroup analysis and interaction results indicate that, within the American population, increases in NEE-CRF are significantly more effective in reducing the risks of hypertension and all-cause mortality in non-diabetic and non-stroke populations compared to diabetic and stroke populations. Diabetic individuals face a higher risk of hypertension due to factors such as insulin resistance, inflammatory responses, and metabolic syndrome (47). Additionally, diabetes, being a metabolic disease, is often accompanied by high insulin levels and hyperglycemia, leading to endothelial dysfunction, which increases the likelihood of hypertension and all-cause mortality (48). For stroke patients, cardiovascular health is often severely compromised post-stroke (49). Many stroke patients may have had undiagnosed hypertension or other cardiovascular diseases prior to the event (50). For instance, chronic hypertension can result in lacunar ischemic strokes due to repeated brain injuries associated with perivascular edema, leading to hyaline deposits, fibrinoid necrosis, thickening of small artery walls, luminal narrowing, and eventually thrombosis and stroke (51). Consequently, the risk of hypertension is generally higher in stroke populations.

Our study has several strengths. Firstly, the two databases we used come from two of the world's largest countries—China and the United States—which exhibit significant differences in factors such as ethnicity and lifestyle. The consistency of findings between these two countries further strengthens our conclusions. Secondly, we not only simultaneously investigated the associations of NEE-CRF with hypertension and all-cause mortality but also employed a complex, multi-stage probability sampling method in analyzing the NHANES database, ensuring that the results are broadly representative of the entire U.S. population.

On the other hand, this study has some limitations. Firstly, the follow-up period of the CHARLS study is relatively short, limiting our ability to assess the long-term impact of NEE-CRF on hypertension and all-cause mortality. Secondly, key variables in this study were based on self-reported data, which may introduce recall bias and social desirability bias. Additionally, differences between the CHARLS and NHANES study samples, including racial, cultural, and lifestyle variations, may affect the applicability of the NEE-CRF equation across diverse populations. Additionally, while our statistical methods were robust, a more detailed standardization process for variables could further enhance the comparability between datasets. Lastly, while our statistical methods were robust, the standardization of variables across datasets could have been more explicitly detailed, which might impact cross-population comparisons.

Despite these limitations, our research demonstrates that NEE-CRF is associated with a lower risk of hypertension and reduced all-cause mortality in two different populations, highlighting its potential clinical significance.

## Conclusion

These findings suggest potential clinical implications, showing a negative correlation between NEE-CRF and both hypertension and all-cause mortality. Based on these results, we recommend considering the regular assessment of NEE-CRF levels in future health monitoring and research. This approach could be valuable for hypertension prevention and may also contribute to improving overall health outcomes.

## Data Availability

Publicly available datasets were analyzed in this study. This data can be found here: availability of data and materials the datasets generated and analyzed during the current study are available in the CHARLS and NHANES website, available in http://charls.pku.edu.cn/en and https://www.cdc.gov/nchs/nhanes/index.htm, respectively.
