# Supplementary material for: The association of non-exercise estimated cardiorespiratory fitness with hypertension and all-cause mortality in American and Chinese populations: evidence from NHANES and CHARLS
Source: Front Cardiovasc Med. 2025 Apr 15;12:1497292. doi: 10.3389/fcvm.2025.1497292 (PMC12037549; doi:10.3389/fcvm.2025.1497292)
Supplement: Supplementary file 1 [file Datasheet1.docx]

**Supplementary Material 1. Detailed information on the CHARLS**

The CHARLS is a comprehensive nationwide population cohort study. The research conducted within the framework of CHARLS has obtained ethical approval from the Biomedical Ethics Review Committee of Peking University (IRB 00001052-11015), and all participants provided their informed consent by signing consent forms. Initiated in 2011 and conducted every two years, CHARLS primarily aims to gather nationally representative data from Chinese individuals aged 45 and above to advance gerontological research. To date, comprehensive data has been available from several phases: the initial baseline survey in 2011, followed by follow-ups in 2013, 2015, 2018, and 2020, along with vital data from the 2014 life course survey. For this study, we selected the baseline data from 2011 and followed participants up until 2020. For the analysis of hypertension as the outcome variable, the screening process was as follows: Initially, 17,708 individuals from the baseline survey conducted between 2011 and 2012 were screened. Participants were excluded if they had no available data for CRF (n = 4,452), had missing data on significant covariates (n = 3,744), were under the age of 45 (n = 189), or had a diagnosis of hypertension at baseline (n = 3,823). Additionally, those with missing hypertension data in 2020 (n = 744) and those lost to follow-up (n = 1,397) were excluded. Ultimately, 3,822 participants were deemed eligible for our study, as illustrated in **Figure S1**. For the analysis of all-cause mortality as the outcome variable, the screening process was as follows: Initially, 17,708 individuals from the baseline survey conducted between 2011 and 2012 were screened. Participants were excluded if they had no available data for CRF (n = 4,452), had missing data on significant covariates (n = 3,763), or were under the age of 45 (n = 189). Additionally, those with missing death data in 2020 (n = 297) and those lost to follow-up (n = 1,399) were excluded. Ultimately, 7,608 participants were deemed eligible for our study, as illustrated in **Figure S2**.


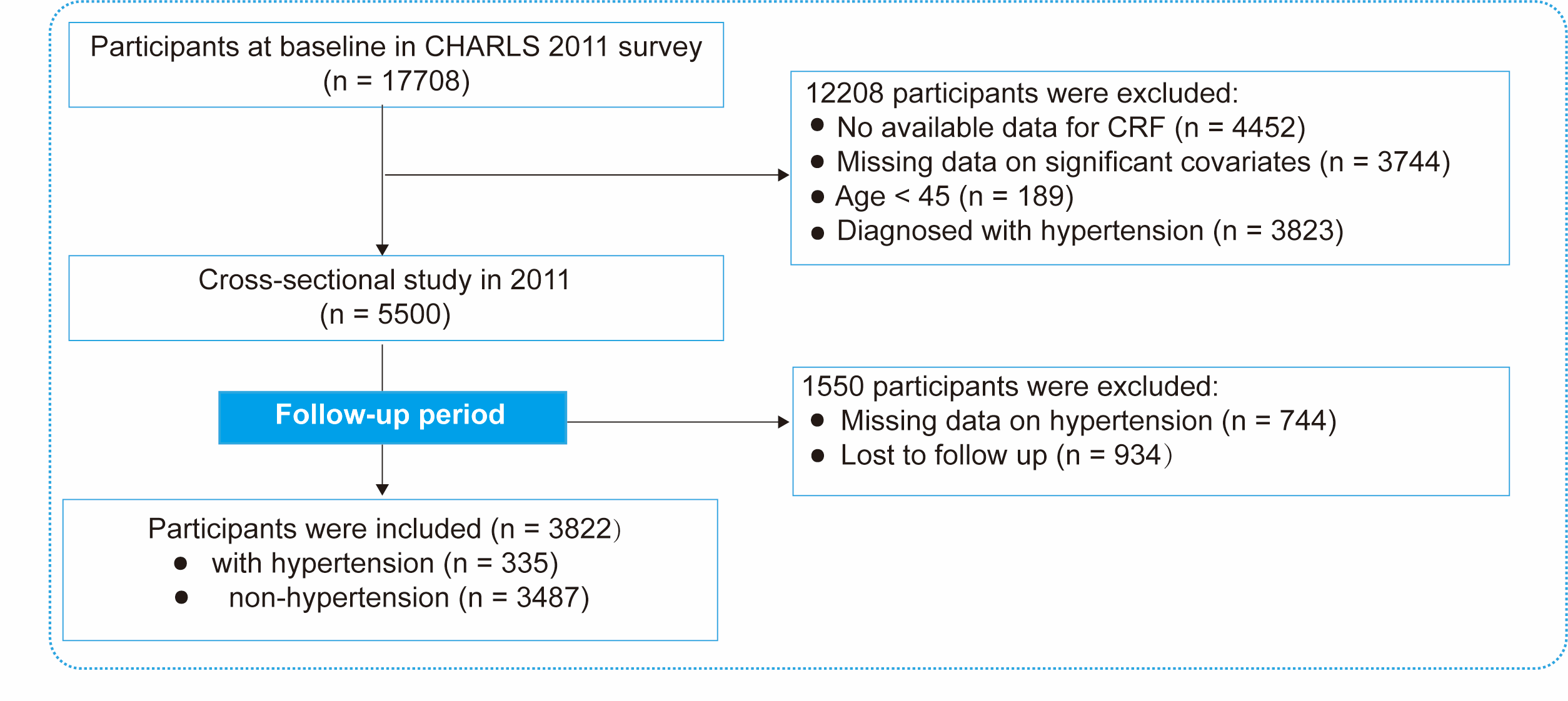


**Figure S1. Flow Chart of the Study Participant Selection Process.**


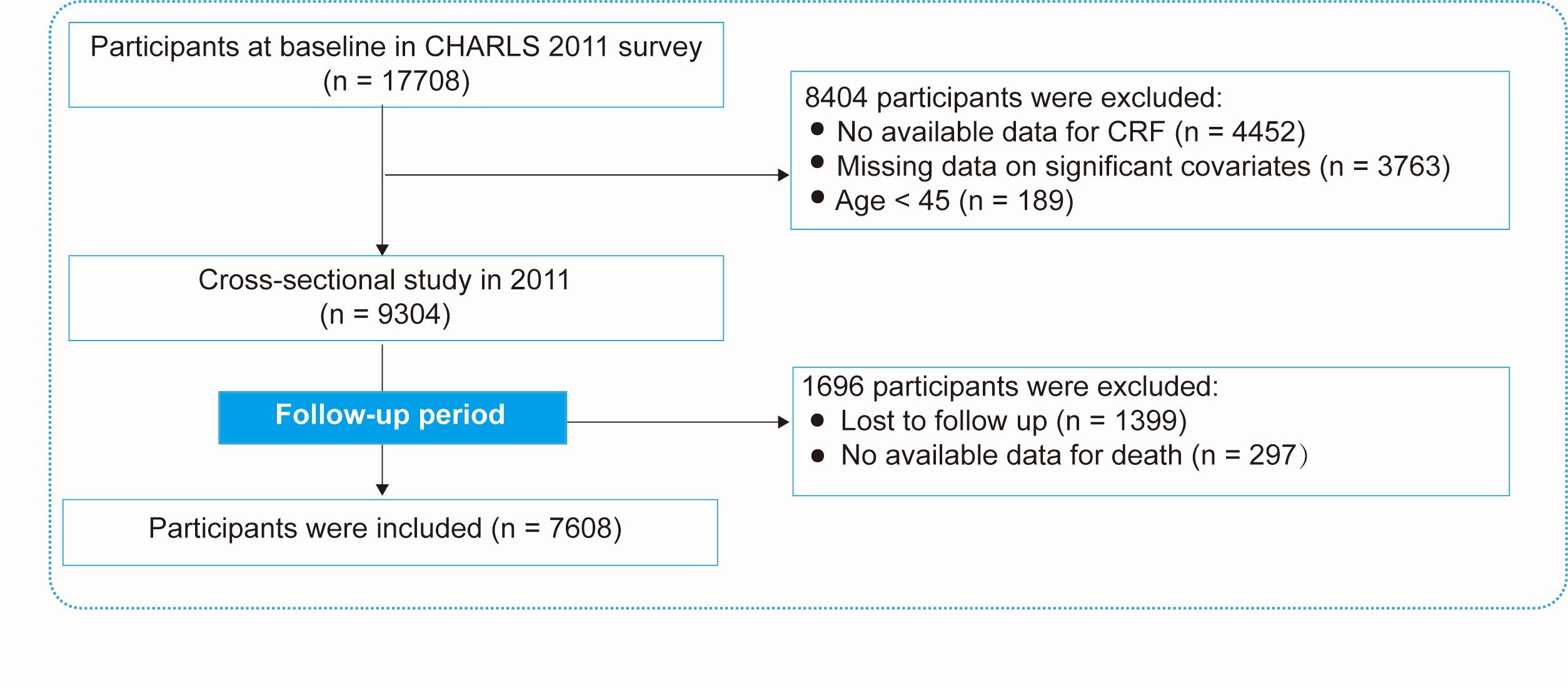


**Figure S2. Flow Chart of the Study Participant Selection Process.**

**Supplementary Material 2. Definition of All-Cause Mortality in CHARLS.**

The mortality rate is determined based on the interview status (alive or deceased) of participants from Wave 2 (2013), Wave 3 (2015), Wave 4 (2018), and Wave 5 (2020). Interview date information is available for all follow-ups, but the exact time of death is only provided in Waves 2 and 5. For deaths with precise records, the interval of survival time is calculated. If no exact death date is available, the median time from the baseline survey date to the participant's death date is used to estimate survival time. If death data is still unavailable, the median time from the first interview date to the wave with recorded deaths is used to estimate survival time.

**Supplementary Material 3. The detailed covariate definitions are used for studying the relationship between NEE-CRF and hypertension using the NHANES.**

| **Covariate** | **Range** | **Grouping for adjustment** |
| --- | --- | --- |
| **Race** | **NA** | Mexican American |
|  |  | Non-Hispanic white |
|  |  | Non-Hispanic black |
|  |  | Other races |
|  |  |  |
| **Education level** | **NA** | Less Than 9th Grade |
|  |  | 9-11th Grade (Includes 12th grade with no diploma) |
|  |  | College Graduate or above |
|  |  | High School Grad/GED or Equivalent |
|  |  | Some college or AA degree |
|  |  |  |
| **Income level [1]** | **PIR: 0.00-5.00 ("5.00" means ≥ 5.00)** | low-income PIR: (0.00-1.00] |
|  |  | middle-income PIR: (1.00-4.00) |
|  |  | high-income PIR: [4.00-5.00] |
|  |  |  |
| **Diabetes [2]** | **NA** | 1.doctor told you have diabetes |
|  |  | 2. glycohemoglobin HbA1c(%) >= 6.5 |
|  |  | 3. fasting glucose (mmol/l) >= 7.0 |
|  |  | 4. random blood glucose (mmol/l) >= 11.1 |
|  |  | 5.two-hour OGTT blood glucose (mmol/l) >= 11.1 |
|  |  | 6.Use of diabetes medication or insulin |
|  |  |  |
| **Marital Status** | **NA** | Divorced |
|  |  | Living with partner |
|  |  | Married |
|  |  | Never married |
|  |  | Separated |
|  |  | Widowed |
|  |  |  |
| **Stroke** | **NA** | “Have you ever been told by a physician or a health professional that you had stroke” |
|  |  | (yes/no) |

**Reference:**

[1] The role of hypertension in bone mineral density among males older than 50 years and postmenopausal females: evidence from the US National Health and Nutrition Examination Survey, 2005-2010 - PubMed. https://pubmed.ncbi.nlm.nih.gov/37397722/. Accessed 25 Oct 2023

[2] Serum selenium concentrations and risk of all-cause and heart disease mortality among individuals with type 2 diabetes - PubMed. https://pubmed.ncbi.nlm.nih.gov/34664061/. Accessed 25 Oct 2023

**Supplementary Material 4. The detailed covariate definitions are used for studying the relationship between NEE-CRF and all-cause mortality using the NHANES.**

The covariates used for all-cause mortality research utilizing the NHANES database include age, sex, ethnicity, marital status, education level, smoking, alcohol consumption, blood glucose, creatinine, serum uric acid, total cholesterol, HDL-C, LDL-C, triglycerides, weight, body mass index, waist circumference, diabetes, and hypertension. Detailed definitions are given in the table below.

| **Covariate** | **Range** | **Grouping for adjustment** |
| --- | --- | --- |
| **Race** | **NA** | Mexican American |
|  |  | Non-Hispanic white |
|  |  | Non-Hispanic black |
|  |  | Other races |
|  |  |  |
| **Education level** | **NA** | Less Than 9th Grade |
|  |  | 9-11th Grade (Includes 12th grade with no diploma) |
|  |  | College Graduate or above |
|  |  | High School Grad/GED or Equivalent |
|  |  | Some college or AA degree |
|  |  |  |
| **Income level [1]** | **PIR: 0.00-5.00 ("5.00" means ≥ 5.00)** | low-income PIR: (0.00-1.00] |
|  |  | middle-income PIR: (1.00-4.00) |
|  |  | high-income PIR: [4.00-5.00] |
|  |  |  |
| **Diabetes [2]** | **NA** | 1.doctor told you have diabetes |
|  |  | 2. glycohemoglobin HbA1c(%) >= 6.5 |
|  |  | 3. fasting glucose (mmol/l) >= 7.0 |
|  |  | 4. random blood glucose (mmol/l) >= 11.1 |
|  |  | 5.two-hour OGTT blood glucose (mmol/l) >= 11.1 |
|  |  | 6.Use of diabetes medication or insulin |
|  |  |  |
| **Marital Status** | **NA** | Divorced |
|  |  | Living with partner |
|  |  | Married |
|  |  | Never married |
|  |  | Separated |
|  |  | Widowed |
|  |  |  |
| **Hypertension [3]** | **NA** | 1. self-reported diagnosis of hypertension |
|  |  | 2.SBP≥140 mmHg and/or DBP≥90 mmHg |
|  |  | 3. the use of antihypertensive medication |

**Reference:**

[1] The role of hypertension in bone mineral density among males older than 50 years and postmenopausal females: evidence from the US National Health and Nutrition Examination Survey, 2005-2010 - PubMed. https://pubmed.ncbi.nlm.nih.gov/37397722/. Accessed 25 Oct 2023

[2] Serum selenium concentrations and risk of all-cause and heart disease mortality among individuals with type 2 diabetes - PubMed. https://pubmed.ncbi.nlm.nih.gov/34664061/. Accessed 25 Oct 2023

[3] Chen L, Zhang J, Zhou N, et al (2023) Association of different obesity patterns with hypertension in US male adults: a cross-sectional study. Sci Rep 13:10551. <https://doi.org/10.1038/s41598-023-37302-x>

**Supplementary Material 5. The detailed covariate definitions are used for studying the relationship between NEE-CRF and hypertension using the CHARLS.**

The covariates used for hypertension research utilizing the CHARLS database include age, sex, residence, marital status, education level,

active physical activity, smoking, alcohol consumption, blood glucose, creatinine, serum uric acid, total cholesterol, HDL-C, LDL-C, triglycerides, weight, body mass index, waist circumference, diabetes, and dyslipidemia. Detailed definitions are given in the table below.

| **Covariate** | **Questionnaire** | **Criteria for classification** |
| --- | --- | --- |
| Diabetes [1] | H_CHARLS + Biochemical data | 1. diagnosis confirmed by a doctor; (2) had fasting blood glucose levels of 126 mg/dL (7.0 mmol/L) or above; (3) had glycosylated hemoglobin levels of 6.5% or above; (4) or were taking medications for diabetes. |
| Education level | What is the highest level of education you have attained? | 1. College and Higher 2. High school 3. Elementary school and below |
| Marital status | What is your marital status? | 1. Married 2. non-married |
| Smoke | H_CHARLS | 1. Former (2) never (3) now |
| Drink | Did you drink any alcoholic beverages last year? | 1. no (2) yes |

**Reference:**

**[1]** Liu Y, Shao J, Liu Q, Zhou W, Huang R, Zhou J, et al. Association between household fuel combustion and diabetes among middle-aged and older adults in China: A cohort study. Ecotoxicol Environ Saf 2023b; 258: 114974.

**Supplementary Material 6. The detailed covariate definitions are used for studying the relationship between NEE-CRF and all-cause mortality using the CHARLS.**

The covariates used for all-cause mortality research utilizing the CHARLS database include age, sex, residence, marital status, education level, active physical activity, smoking, alcohol consumption, blood glucose, creatinine, serum uric acid, total cholesterol, HDL-C, LDL-C, triglycerides, weight, body mass index, waist circumference, hypertension, diabetes, and dyslipidemia. Detailed definitions are given in the table below.

| **Covariate** | **Questionnaire** | **Criteria for classification** |
| --- | --- | --- |
| Hypertension [1] | H_CHARLS + Biochemical data | (1) a systolic blood pressure of 140 mmHg or more; (2) a diastolic blood pressure of 90 mmHg or more; (3) a self-reported history of hypertension; or (4) the use of medication to lower blood pressure |
| Diabetes [2] | H_CHARLS + Biochemical data | 1. diagnosis confirmed by a doctor; (2) had fasting blood glucose levels of 126 mg/dL (7.0 mmol/L) or above; (3) had glycosylated hemoglobin levels of 6.5% or above; (4) or were taking medications for diabetes. |
| Education level | What is the highest level of education you have attained? | 1. College and Higher 2. High school 3. Elementary school and below |
| Marital status | What is your marital status? | 1. Married 2. non-married |
| Smoke | H_CHARLS | 1. Former (2) never (3) now |
| Drink | Did you drink any alcoholic beverages last year? | 1. no (2) yes |

**Reference:**

**[1]** Luo JH, Zhang TM, Yang LL, Cai YY, Yang Y. Association between relative muscle strength and hypertension in middle-aged and older Chinese adults. BMC Public Health 2023; 23: 2087.

**[2]** Liu Y, Shao J, Liu Q, Zhou W, Huang R, Zhou J, et al. Association between household fuel combustion and diabetes among middle-aged and older adults in China: A cohort study. Ecotoxicol Environ Saf 2023b; 258: 114974.

**Supplementary Material 7. Population Characteristics Classified by Hypertension Status in China.**

Supplementary Material 7 presents the baseline characteristics of participants stratified by hypertension status. Using 2011 as the baseline and following up until 2020, the 9-year follow-up data revealed that there were 335 participants in the hypertension group and 3,487 in the non-hypertension group. The average age of the hypertension group was 56.96 ± 8.22 years, with 42.69% male and 57.31% female. The NEE-CRF value for this group was 10.13 ± 1.89, and the resting heart rate (rHR) was 72.89 ± 10.29 bpm. In contrast, the non-hypertension group had an average age of 56.35 ± 7.96 years, with 45.68% male and 54.32% female. The NEE-CRF value for this group was 10.57 ± 1.92, and the rHR was 71.69 ± 10.05 bpm. Compared to the non-hypertension group, the hypertension group had significantly higher values for age, body mass index (BMI), weight, waist circumference, total cholesterol (TC), triglycerides (TG), blood glucose, serum uric acid, and both systolic blood pressure (SBP) and diastolic blood pressure (DBP), with statistically significant differences (*P* < 0.001).

| **Variable** | **Total (n=3822)** | **Non-Hypertension (n=3487)** | **Hypertension (n=335)** | **P-value** |
| --- | --- | --- | --- | --- |
| **Age (years)** | 56.40 ± 7.99 | 56.35 ± 7.96 | 56.96 ± 8.22 | 0.20 |
| **Glucose (mg/dL)** | 104.98 ± 26.55 | 104.54 ± 24.94 | 109.50 ± 39.37 | **0.02** |
| **Creatinine (mg/dL)** | 0.76 ± 0.17 | 0.76 ± 0.16 | 0.76 ± 0.18 | 0.79 |
| **SUA (mg/dL)** | 4.27 ± 1.16 | 4.27 ± 1.15 | 4.35 ± 1.19 | 0.22 |
| **TC (mg/dL)** | 190.72 ± 36.68 | 190.18 ± 36.21 | 196.31 ± 40.86 | **<0.01** |
| **HDL-C (mg/dL)** | 52.56 ± 14.94 | 52.65 ± 14.90 | 51.62 ± 15.30 | 0.24 |
| **LDL-C (mg/dL)** | 115.33 ± 33.04 | 115.07 ± 32.82 | 118.06 ± 35.22 | 0.14 |
| **TG (mg/dL)** | 119.34 ± 86.23 | 118.03 ± 85.49 | 132.97 ± 92.63 | **<0.01** |
| **Height (m)** | 1.58 ± 0.08 | 1.58 ± 0.08 | 1.57 ± 0.08 | 0.11 |
| **Weight (kg)** | 57.05 ± 10.44 | 56.88 ± 10.42 | 58.89 ± 10.43 | **<0.001** |
| **BMI (kg/m^2^)** | 22.77 ± 3.44 | 22.68 ± 3.43 | 23.71 ± 3.39 | **<0.001** |
| **Waist Circumference (cm)** | 81.54 ± 11.85 | 81.29 ± 11.82 | 84.05 ± 11.90 | **<0.001** |
| **SBP (mmHg)** | 116.85 ± 11.33 | 116.45 ± 11.33 | 121.03 ± 10.52 | **<0.001** |
| **DBP (mmHg)** | 69.81 ± 8.69 | 69.57 ± 8.69 | 72.34 ± 8.35 | **<0.001** |
| **rHR (bpm)** | 71.79 ± 10.07 | 71.69 ± 10.05 | 72.89 ± 10.29 | **0.04** |
| **NEE-CRF (METs)** | 10.53 ± 1.92 | 10.57 ± 1.92 | 10.13 ± 1.89 | **<0.001** |
|  |  |  |  |  |
| **Sex, n(%)** |  |  |  | 0.32 |
| Female | 2086(54.58) | 1894(54.32) | 192(57.31) |  |
| Male | 1736(45.42) | 1593(45.68) | 143(42.69) |  |
|  |  |  |  |  |
| **Marital status, n(%)** |  |  |  | 0.23 |
| Married | 3526(92.26) | 3223(92.43) | 303(90.45) |  |
| Non-married | 296(7.74) | 264(7.57) | 32(9.55) |  |
|  |  |  |  |  |
| **Active Physical activity, n(%)** |  |  |  | 0.05 |
| No | 2742(71.74) | 2486(71.29) | 256(76.42) |  |
| Yes | 1080(28.26) | 1001(28.71) | 79(23.58) |  |
|  |  |  |  |  |
| **Education, n(%)** |  |  |  | **<0.01** |
| College and Higher | 49(1.28) | 49(1.41) | 0(0.00) |  |
| Elementary school and below | 2537(66.38) | 2294(65.79) | 243(72.54) |  |
| High school | 1236(32.34) | 1144(32.81) | 92(27.46) |  |
|  |  |  |  |  |
| **Residence, n(%)** |  |  |  | 0.13 |
| Rural | 2602(68.08) | 2361(67.71) | 241(71.94) |  |
| Urban | 1220(31.92) | 1126(32.29) | 94(28.06) |  |
|  |  |  |  |  |
| **Smoke, n(%)** |  |  |  | 0.37 |
| Former | 273(7.14) | 248(7.11) | 25(7.46) |  |
| Never | 2376(62.17) | 2156(61.83) | 220(65.67) |  |
| Now | 1173(30.69) | 1083(31.06) | 90(26.87) |  |
|  |  |  |  |  |
| **Drink, n(%)** |  |  |  | 0.63 |
| No | 2516(65.83) | 2300(65.96) | 216(64.48) |  |
| Yes | 1306(34.17) | 1187(34.04) | 119(35.52) |  |
|  |  |  |  |  |
| **DM, n(%)** |  |  |  | **<0.01** |
| No | 3447(90.19) | 3162(90.68) | 285(85.07) |  |
| Yes | 375(9.81) | 325(9.32) | 50(14.93) |  |

**Notes:** All values are presented as proportion (%) for categorical variables, assessed via weighted chi-square tests, or mean (standard deviation) for continuous variables, assessed via weighted Student’s t-tests.

**Abbreviations**: **NEE-CRF:** non-exercise estimated cardiorespiratory fitness; **rHR**: resting heart rate; **SBP**: systolic blood pressure; **DBP**: diastolic blood pressure; **SUA**: serum uric acid; **TG**: triglyceride; **TC:** total cholesterol; **HDL-C:** high density lipoprotein cholesterol; **LDL-C:** low density lipoprotein cholesterol; **DM**: diabetes mellitus

**Supplementary Material 8. Population Characteristics Classified by NEE-CRF Four Categories in China.**

As shown in Supplementary Material 8, the quartile ranges of NEE-CRF are 0.41-8.66, 8.66-9.76, 9.76-11.64, and 11.64-15.88. Additionally, we observed that compared to participants with lower NEE-CRF, those in the fourth quartile (Q4) had significantly lower age, rHR, BMI, waist circumference, TC, and HDL-C (P < 0.001).

|  | **Cardiorespiratory fitness** | | | | | |
| --- | --- | --- | --- | --- | --- | --- |
| **Characteristics** | **Total** | **Q1** | **Q2** | **Q3** | **Q4** | ***P*-value** |
|  | **(0.41-15.88)** | **(0.41-8.66)** | **(8.66-9.76)** | **(9.76-11.64)** | **(11.64-15.88)** |  |
| **Age (years)** | 58.35 ± 8.66 | 63.69 ± 9.09 | 56.33 ± 7.63 | 57.71 ± 8.66 | 55.67 ± 6.66 | **<0.001** |
| **Glucose (mg/dL)** | 109.25 ± 33.86 | 113.78 ± 37.99 | 108.46 ± 31.94 | 109.65 ± 35.03 | 105.11 ± 29.33 | **<0.001** |
| **Creatinine (mg/dL)** | 0.77 ± 0.19 | 0.72 ± 0.16 | 0.71 ± 0.16 | 0.81 ± 0.24 | 0.85 ± 0.16 | **<0.001** |
| **SUA (mg/dL)** | 4.41 ± 1.22 | 4.26 ± 1.15 | 4.04 ± 1.11 | 4.55 ± 1.34 | 4.78 ± 1.16 | **<0.001** |
| **TC (mg/dL)** | 193.98 ± 38.05 | 202.90 ± 39.25 | 196.32 ± 37.69 | 190.31 ± 35.98 | 186.39 ± 37.16 | **<0.001** |
| **HDL-C (mg/dL)** | 51.24 ± 15.13 | 49.12 ± 14.05 | 51.09 ± 14.22 | 51.07 ± 15.43 | 53.69 ± 16.37 | **<0.001** |
| **LDL-C (mg/dL)** | 117.00 ± 34.92 | 124.28 ± 36.75 | 118.85 ± 34.67 | 113.88 ± 33.00 | 111.00 ± 33.69 | **<0.001** |
| **TG (mg/dL)** | 131.46 ± 95.69 | 148.65 ± 94.55 | 135.90 ± 95.97 | 129.56 ± 104.33 | 111.71 ± 83.04 | **<0.001** |
| **Height (m)** | 1.58 ± 0.08 | 1.52 ± 0.07 | 1.55 ± 0.07 | 1.60 ± 0.08 | 1.64 ± 0.07 | **<0.001** |
| **Weight (kg)** | 58.94 ± 11.37 | 60.76 ± 13.18 | 57.37 ± 10.67 | 59.58 ± 12.39 | 58.07 ± 8.34 | **<0.001** |
| **BMI (kg/m^2^)** | 23.59 ± 3.82 | 26.00 ± 4.61 | 23.80 ± 3.17 | 23.05 ± 3.29 | 21.51 ± 2.39 | **<0.001** |
| **Waist (cm)** | 84.22 ± 12.65 | 90.67 ± 11.87 | 84.10 ± 11.73 | 83.36 ± 12.79 | 78.76 ± 11.25 | **<0.001** |
| **rHR (bpm)** | 72.13 ± 10.55 | 75.97 ± 10.74 | 71.93 ± 9.56 | 71.10 ± 10.66 | 69.51 ± 10.10 | **<0.001** |
| **SBP (mmHg)** | 129.43 ± 20.79 | 136.30 ± 21.79 | 127.95 ± 21.10 | 128.32 ± 20.00 | 125.15 ± 18.44 | **<0.001** |
| **DBP (mmHg)** | 75.50 ± 11.91 | 77.09 ± 11.51 | 75.21 ± 11.76 | 75.43 ± 12.21 | 74.29 ± 11.99 | **<0.001** |
|  |  |  |  |  |  |  |
| **Active Physical activity, n(%)** |  |  |  |  |  | **<0.001** |
| No | 5625(73.94) | 1674(88.01) | 1412(74.24) | 1313(69.03) | 1226(64.46) |  |
| Yes | 1983(26.06) | 228(11.99) | 490(25.76) | 589(30.97) | 676(35.54) |  |
|  |  |  |  |  |  |  |
| **Sex, n(%)** |  |  |  |  |  | **<0.001** |
| Female | 4202(55.23) | 1806(94.95) | 1663(87.43) | 730(38.38) | 3(0.16) |  |
| Male | 3406(44.77) | 96(5.05) | 239(12.57) | 1172(61.62) | 1899(99.84) |  |
|  |  |  |  |  |  |  |
| **Marital Status, n(%)** |  |  |  |  |  | **<0.001** |
| Married | 6802(89.41) | 1529(80.39) | 1735(91.22) | 1772(93.17) | 1766(92.85) |  |
| Non-Married | 806(10.59) | 373(19.61) | 167(8.78) | 130(6.83) | 136(7.15) |  |
|  |  |  |  |  |  |  |
| **Education, n(%)** |  |  |  |  |  | **<0.001** |
| College and higher | 93(1.22) | 5( 0.26) | 24(1.26) | 39(2.05) | 25(1.31) |  |
| Elementary school and below | 5306(69.74) | 1589(83.54) | 1404(73.82) | 1233(64.83) | 1080(56.78) |  |
| High school | 2209(29.04) | 308(16.19) | 474(24.92) | 630(33.12) | 797(41.90) |  |
|  |  |  |  |  |  |  |
| **Residence, n(%)** |  |  |  |  |  | **<0.001** |
| Rural | 5096(66.98) | 1185(62.30) | 1288(67.72) | 1243(65.35) | 1380(72.56) |  |
| Urban | 2512(33.02) | 717(37.70) | 614(32.28) | 659(34.65) | 522(27.44) |  |
|  |  |  |  |  |  |  |
| **Smoke, n(%)** |  |  |  |  |  | **<0.001** |
| Current smoker | 2241(29.46) | 178(9.36) | 185(9.73) | 687(36.12) | 1191(62.62) |  |
| Former smoker | 610(8.02) | 90(4.73) | 77(4.05) | 237(12.46) | 206(10.83) |  |
| Never | 4757(62.53) | 1634(85.91) | 1640(86.23) | 978(51.42) | 505(26.55) |  |
|  |  |  |  |  |  |  |
| **Drink, n(%)** |  |  |  |  |  | **<0.001** |
| no | 5130(67.43) | 1664(87.49) | 1564(82.23) | 1137(59.78) | 765(40.22) |  |
| yes | 2478(32.57) | 238(12.51) | 338(17.77) | 765(40.22) | 1137(59.78) |  |
|  |  |  |  |  |  |  |
| **Hypertension, n(%)** |  |  |  |  |  | **<0.001** |
| no | 4641(61.00) | 830(43.64) | 1207(63.46) | 1204(63.30) | 1400(73.61) |  |
| yes | 2967(39.00) | 1072(56.36) | 695(36.54) | 698(36.70) | 502(26.39) |  |
|  |  |  |  |  |  |  |
| **DM, n(%)** |  |  |  |  |  | **<0.001** |
| no | 6552(86.12) | 1539(80.91) | 1655(87.01) | 1636(86.01) | 1722(90.54) |  |
| yes | 1056(13.88) | 363(19.09) | 247(12.99) | 266(13.99) | 180(9.46) |  |
|  |  |  |  |  |  |  |
| **Dyslipidemia, n(%)** |  |  |  |  |  | **<0.001** |
| no | 4532(59.57) | 926(48.69) | 1141(59.99) | 1142(60.04) | 1323(69.56) |  |
| yes | 3076(40.43) | 976(51.31) | 761(40.01) | 760(39.96) | 579(30.44) |  |
|  |  |  |  |  |  |  |
| **Death, n(%)** |  |  |  |  |  | **<0.001** |
| No | 7440(97.79) | 1832(96.32) | 1880(98.84) | 1855(97.53) | 1873(98.48) |  |
| Yes | 168(2.21) | 70(3.68) | 22(1.16) | 47(2.47) | 29(1.52) |  |

**Notes:** All values are presented as proportion (%) for categorical variables, assessed via weighted chi-square tests, or mean (standard deviation) for continuous variables, assessed via weighted Student’s t-tests.

**Abbreviations**: **NEE-CRF:** non-exercise estimated cardiorespiratory fitness; **rHR**: resting heart rate; **SBP**: systolic blood pressure; **DBP**: diastolic blood pressure; **SUA**: serum uric acid; **TG**: triglyceride; **TC:** total cholesterol; **HDL-C:** high density lipoprotein cholesterol; **LDL-C:** low density lipoprotein cholesterol; **DM**: diabetes mellitus

**Supplementary Material 9. Associations of NEE-CRF with Hypertension in Chinese Population.**

| **Hypertension** | **OR^a^ (95% CI), *P*-value** | | |
| --- | --- | --- | --- |
|  | **Crude model ^b^** | **Model 1^c^** | **Model 2^d^** |
| **Continuous** | 0.89 (0.83,0.94) **<0.001** | 0.89 (0.84, 0.95) **<0.001** | 0.87 (0.81, 0.94) **<0.001** |
| **Categorical** |  |  |  |
| **Q1** | **Reference** | **Reference** | **Reference** |
| **Q2** | 0.60 (0.44,0.82) **<0.001** | 0.62 (0.45, 0.85) **0.003** | 0.63 (0.46, 0.87) **0.005** |
| **Q3** | 0.63 (0.46,0.85) **0.003** | 0.67(0.49, 0.92) **0.01** | 0.63 (0.45, 0.88) **0.01** |
| **Q4** | 0.54 (0.40,0.74) **<0.001** | 0.57(0.40, 0.79) **0.001** | 0.52 (0.36, 0.75) **<0.001** |
| ***P* for trend** | **<0.001** | **0.004** | **<0.001** |
|  |  |  |  |

**Notes**: In sensitivity analysis, NEE-CRF is transformed from a continuous variable to a categorical variable (Quartiles); **OR^a^**: effect size**; Crude model ^b^**: no covariates were adjusted; **Model 1^c^:** adjusted for education level and marital status; **Model 2^d^:** adjusted for education level, marital status, blood glucose, creatinine, serum uric acid, total cholesterol, triglyceride, HDL-C, LDL-C, alcohol consumption, and diabetes

**Abbreviations**: **NEE-CRF:** non-exercise estimated cardiorespiratory fitness; **95% CI**: 95% confidence interval; **OR**: odds ratio.

**Supplementary Material 10. Associations of NEE-CRF with All-Cause Mortality in Chinese Population.**

| **All-cause mortality** | **HR^a^ (95% CI), *P*-value** | | |
| --- | --- | --- | --- |
|  | **Crude model ^b^** | **Model 1^c^** | **Model 2^d^** |
| **Continuous** | 0.83 (0.76,0.89) **<0.001** | 0.91 (0.84, 0.98) **0.02** | 0.88 (0.80, 0.96) **0.003** |
| **Categorical** |  |  |  |
| **Q1** | **Reference** | **Reference** | **Reference** |
| **Q2** | 0.31 (0.19,0.50) **<0.001** | 0.40 (0.25, 0.66) **<0.001** | 0.39 (0.24, 0.64) **<0.001** |
| **Q3** | 0.67 (0.46,0.97) **0.03** | 0.96(0.65, 1.40) 0.81 | 0.87(0.58, 1.30) 0.50 |
| **Q4** | 0.41 (0.27,0.63) **<0.001** | 0.62(0.40, 0.98) **0.04** | 0.55(0.34, 0.90) **0.02** |
| ***P* for trend** | **<0.001** | 0.20 | 0.08 |
|  |  |  |  |

**Notes**: In sensitivity analysis, NEE-CRF is transformed from a continuous variable to a categorical variable (Quartiles); **HR^a^**: effect size**; Crude model ^b^**: no covariates were adjusted; **Model 1^c^:** adjusted for education level and marital status; **Model 2^d^:** adjusted for education level, marital status, blood glucose, creatinine, serum uric acid, total cholesterol, triglyceride, HDL-C, LDL-C, alcohol consumption, and diabetes

**Abbreviations**: **NEE-CRF:** non-exercise estimated cardiorespiratory fitness; **95% CI**: 95% confidence interval; **HR**: hazard ratio.

**Supplementary Material 11. Subgroup Analysis of the Association Between NEE-CRF and Hypertension and All-Cause Mortality in the American Population**


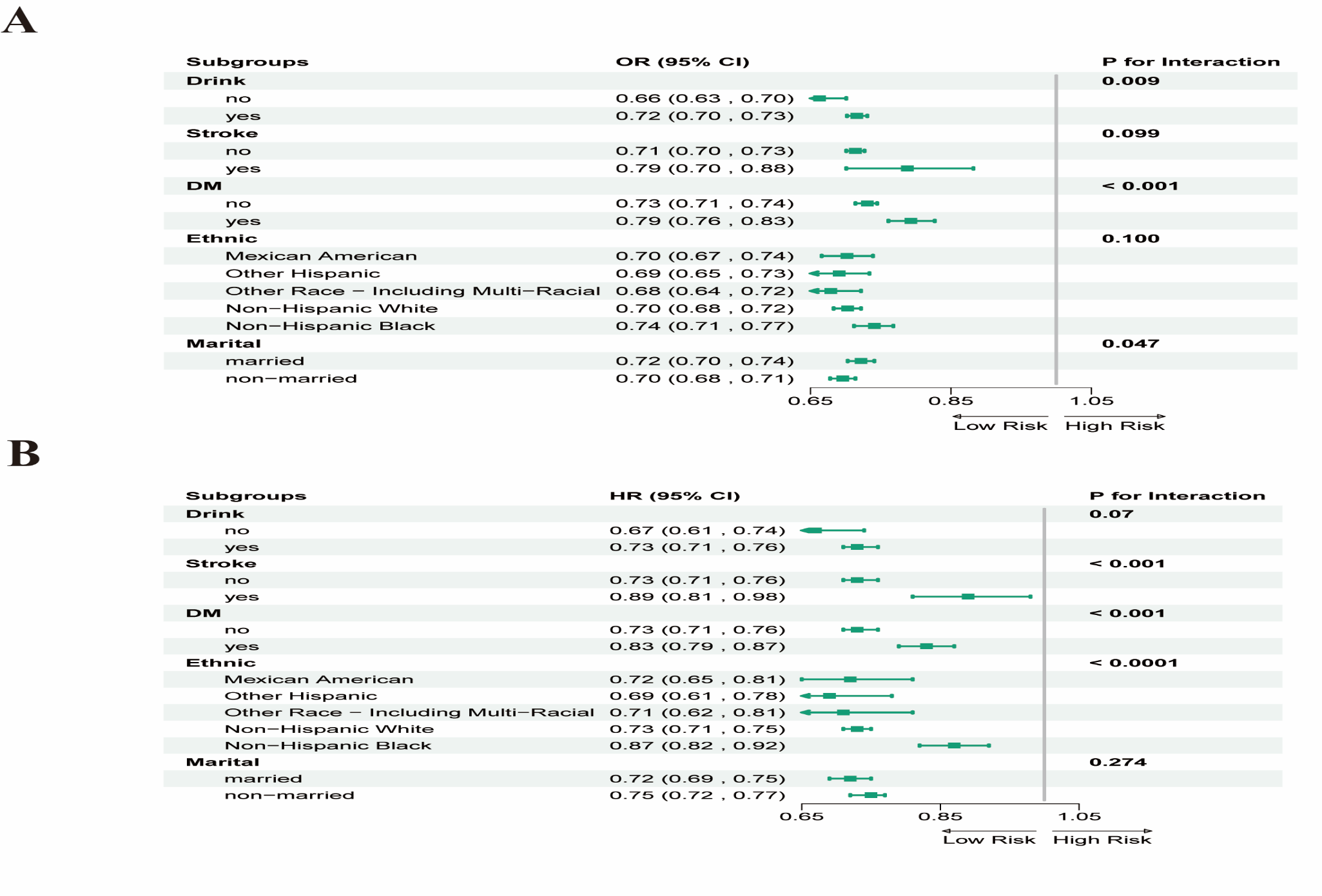


**Supplementary Material 12. Subgroup Analysis of the Association Between NEE-CRF and Hypertension and All-Cause Mortality in the Chinese Population.**

**
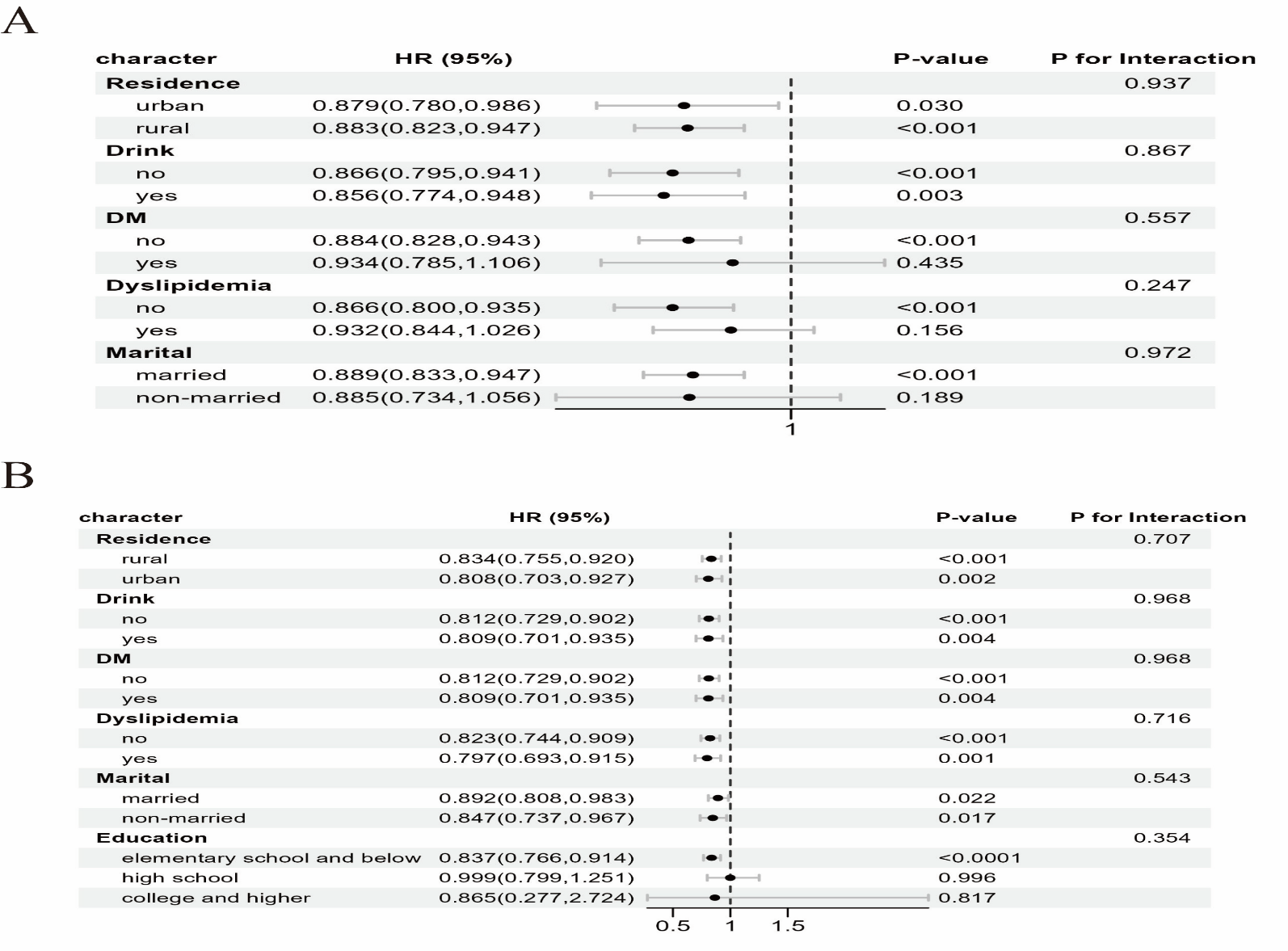
**

**Supplementary Material 13. Smoothed curve fitting: Dose-response relationship between NEE-CRF with Hypertension and All-Cause Mortality in Chinese Population.**

.


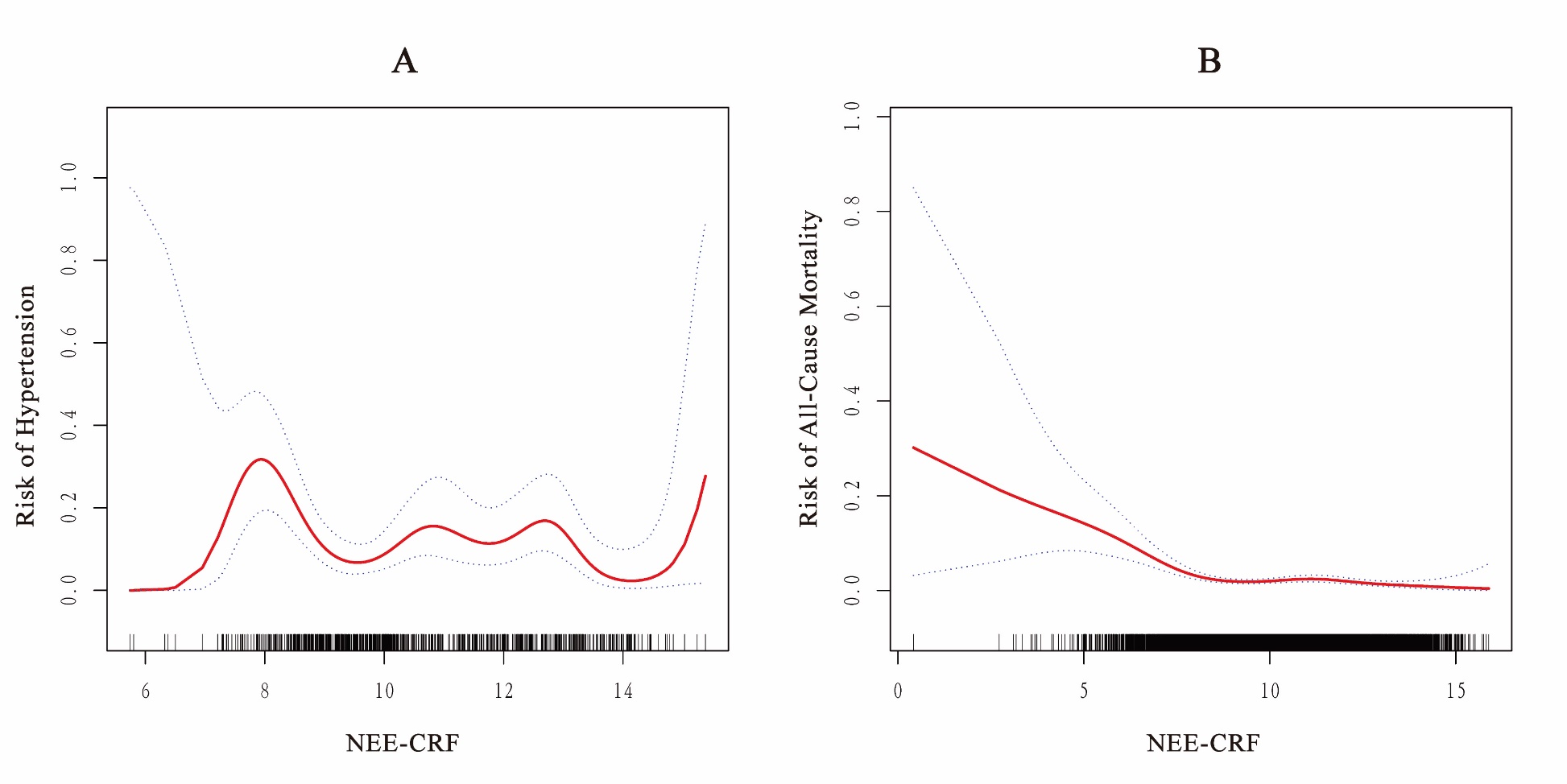


**Supplementary Material 14. Threshold effect analysis of NEE-CRF with Hypertension and All-Cause Mortality using two-piecewise linear regression model in Chinese Population.**

| **All-Cause Mortality** | **Adjust HR (95% CI) P value** |
| --- | --- |
| **Fitting by linear regression model** | 0.84 (0.76, 0.91) <0.001 |
| **Fitting by two-piecewise Cox proportional risk model** |  |
| **Inflection point** | 8.74 |
| <8.74 | 0.66 (0.56, 0.79) <0.001 |
| >8.74 | 0.96 (0.85, 1.09) 0.51 |
| **Log likelihood ratio test** | 0.005 |
| **Hypertension** | **Adjust OR (95% CI) P value** |
| **Fitting by linear regression model** | 0.90 (0.80, 1.00) 0.06 |
| **Fitting by two-piecewise linear regression model** |  |
| **Inflection point** | 9.40 |
| <9.40 | 0.60 (0.40, 0.90) 0.03 |
| >9.40 | 1.00 (0.80, 1.20) 0.80 |
| **Log likelihood ratio test** | 0.10 |

**Note**: Adjusted for education level, marital status, blood glucose, creatinine, serum uric acid, total cholesterol, triglyceride, HDL-C, LDL-C, alcohol consumption, and diabetes.

**Abbreviations**: **NEE-CRF:** non-exercise estimated cardiorespiratory fitness; **95% CI**: 95% confidence interval; **OR**: odds ratio; **HR**: hazard ratio
